# Dr. Blundell's Physiological and Pathological Researches

**Published:** 1825-04-01

**Authors:** 


*Researches, Physiological and Pathological; instituted principally with a View to the Improvement of Medical and Surgical Practice.* By JAMES BLUNDELL, M.D. Lecturer on Physiology and Midwifery at the united Hospitals of St. Thomas and Guy. Octavo, pp. 146, two Plates. Cox and Son, Borough, December, 1824.

PHYSIOLOGICAL experiments are much more rare in this country than on the continent—especially France. The reason seems to be that, in the latter country, physiologists make experiments for the purpose of discovering some object—while here, our physiologists experiment with a direct, and generally useful object in view, and in the hope of attaining that object. This last course is, we imagine, the only laudable and legitimate one which we ought to pursue. We do not hold man so high, nor brutes so low, in the scale of created things, as to imagine that the feelings and the lives of the *latter* should be wantonly expended in satisfying the mere *curiosity* of the *former.* It is true that animals must die to feed man, or man himself might perish; but it must be remembered, that death in *this* way is a very different kind of thing from death by *physiological experiment.* The guillotine and the Indian torture are somewhat different *modes* of coming to the same result!

Dr. Blundell has long been known as a zealous cultivator of the science he professes; and not only a zealous, but an able one. The first part of the volume before us consists of a paper which was read in the Medico-Chirurgical Society about two winters ago, but not published—for what reason is only known to "the Committee of Public Safety," in their aggregate of wisdom and plenitude of power assembled.

Some of our author's experiments certainly appear somewhat equivocal in respect to the *cui bono;* but still it must be conceded that there is always an object in view, physiological or pathological; though that object does not always seem in close correspondence with a directly useful or practical purpose. Dr. B. observes that, of all the branches of surgery, there is none which admits of greater improvement than the surgery of the abdomen. Under this impression, he has been induced to accumulate the facts and observations contained in the Essay under consideration. We shall take a rapid coup d'œil of the principal experiments here brought forward.

From four rabbits, Dr. B. extirpated the left kidney, through an incision about an inch in length, the kidney being drawn up through the wound, and the superior half of the peritoneal attachment, thus put on the stretch, included with it in the ligature. All these animals died—two from the inflammation supervening on the operation—one from the formation of a sac in the place where the kidney had previously lodged—and one from cause unascertained.

In seven experiments our author removed the spleen. The two first rabbits died in a few days, of peritoneal inflammation—the third survived three months, but ultimately died of diffused peritonitis and the formation of a sac in the site of the spleen. The 4th died in a few days, of purging and peritonitis—the 5th recovered for a time, but at the end of six months began to pine, and died at last of abdominal inflammation. The 6th and 7th rabbits recovered permanently.

In five rabbits he opened the abdominal cavity, over the bladder, in the course of the linea alba—punctured the fundus vesicæ with a lancet, and secured the aperture by ligature. Of these, three recovered completely—and two died, one of them 14 days after the operation, the other 17 days. In two rabbits, Dr. Blundell cut off at least one quarter of the bladder at the fundus, by one stroke of the scissors, a ligature having been previously applied. One of them lived seven months, and then died of the purulent sacs already mentioned. The second survived, and still lives, fat and healthy.

Into the peritoneal cavities of four rabbits, our author injected about an ounce of human urine—left it there an hour, and then discharged it, washing out the peritoneum thoroughly with tepid water. They all suffered much collapse from the experiment, but seemed soothed by the injection of tepid water. Of these, the first sunk under the primary impression of the operation—the second died in sixty hours from peritoneal inflammation—the third died from diffused and very active peritonitis in nineteen hours (the urine having been very negligently washed out)—the fourth rabbit recovered completely.

Of seven rabbits, into whose abdomens the *decoctum quercus* was injected, six died and one recovered.

The peritoneal inflammation, in all the more decisive cases, was marked by serous effusion—accumulation of adhesive matter—agglutination of the viscera—and, in some instances, by so thorough an injection of the capillary vessels, as to produce a petechial appearance.

From the facts ascertained by the preceding experiments, the following inferences are drawn by Dr. Blundell.

"1st. Large apertures into the peritoneum of the rabbit do not immediately induce a dangerous prostration of strength. In all my experiments, I never once observed any marked collapse in the animal at the moment when the peritoneum was laid open, though I was in full expectation of it. When urine was injected, collapse was immediately and evidently produced.

"2ly. Large apertures into the peritoneal sac, in the rabbit, are not necessarily, nor perhaps generally productive of fatal inflammation. Of eighteen rabbits, not only opened, but subjected to further violence, five only died from this cause.

"3ly. In the rabbit, the kidney, the spleen, and a large piece of the bladder may be extirpated without necessarily causing death; though death under the first operation is probable.

"4ly. When the abdomen is laid open, and parts are removed from it in the rabbit, the first danger arises apparently from collapse, the second from general inflammation, and the last from chronic topical disease.

"5ly. The rabbit's abdomen is very tender, probably no less so than that of man. Of twenty-nine rabbits, twenty-one died more or less directly from the operations performed, some of them, it must be confessed, violent ones; and it should be observed particularly, that five, out of seven rabbits, died from the splenic operation carefully performed; though both cases, hereafter recorded, in which the human spleen was removed, under circumstances to appearance highly unfavourable, terminated in complete and uninterrupted recovery. The general impression left on my mind by many observations is, that the abdomen of the rabbit is, on the whole, no less tender than the human.

"6ly. It follows, from the former inference, that success in abdominal operations on the rabbit, furnishes a presumption in favour of success in similar operations on the human abdomen; and therefore, from these experiments, we may infer *presumptively* that moderate openings into the human peritoneum will not necessarily, nor even generally, prove fatal from inflammation or otherwise; and, further, that certain viscera or parts of viscera, not essential to the welfare of our structure, may be removed from the belly without necessarily, or even generally producing death. The extirpation of the kidney must be highly dangerous; but there is a presumption in favour of the successful removal of the spleen, the ovaries, or even of large pieces of the bladder." 12.

From these *presumptions,* drawn from experiments on the rabbit, our author proceeds to what history teaches us respecting the human frame itself. Of the slighter injuries of the abdomen, resulting from tapping, slight wounds (not penetrating the intestines)—hernial operations—Pott's case of extirpation of the ovaries, &c. our author does not think it necessary to take notice, as they are familiar examples. But—

"Of severer injuries of the abdomen, with their results, the following may be adduced as having, with few exceptions, fallen under my own notice, or that of my friends; and, as possessing an authenticity, on which, where there is no observation to the contrary, I can thoroughly rely. These, as it will be perceived, so far as they furnish inferences at all, confirm those taken from experiments on the rabbit; and form, apparently, a part of one harmonious system of facts, which mutually support each other.

"1st. One case, the only one I know of, in which the mouth of the womb was torn off, and came completely away; large bleeding and collapse were produced, but the patient recovered. My friend, Mr. Scott of Norwich, carefully investigated this case, and Dr. Merriman of London is now, I believe, in possession of the preparation.

"2dly. One case, in which, from defective formation of the external genitals, the child's head could not readily pass: it forced its way into the rectum, and was born at the anus, occasioning three large rents, two laterally, and one forward; the woman recovered without any very pressing symptom. Mr. Harrison of Greenwich, had the woman ultimately under his own care; and himself, in conjunction with my friend Mr. Gaitskell, obliged me with the relation of it.

"3dly. Four cases of chronic inversion of the womb, in which the uterus was extirpated by ligature, at different ages.

"One, a case under the care of Mr. Chevalier. The woman in this instance was, I think, about sixty; and for years previously had ceased to menstruate: no bad symptoms seem to have occurred.

"The second, a case under the care of Mr. Newnham. The woman was about twenty-four. Some difficulties arose from the extreme irritability of the patient, but the greater part of the womb was got away. The preparation of the womb I saw myself. The woman is still doing well, and it is now six or seven years since the operation was performed.

"The third, a case in which Dr. Hull of Manchester operated. The patient was excessively irritable and intractable, and some difficulties occurred as in the former instance, but the operation succeeded. Dr. Hull himself related this case to me in a conversation between us.

"The fourth, a case of my own, in which the greater part of the womb was removed by a wire ligature. It came away in eleven days. This patient was of a tranquil torpid habit; and not one bad symptom occurred.

"4thly. One case has fallen under my observation, in which a fall from the top of a coach occasioned a transverse rent through the abdominal coverings, above the abdominal rings, on the right side, four fingers broad at the least. The intestines hung out. The man recovered in a few weeks. The intestines still protrude at this part, pushing the abdominal coverings before them, and forming a ventral hernia. The scar of the rent is still apparent. The man was under the care of Mr. Green, one of the surgeons of St. Thomas's Hospital.

"5thly. Two cases, (may be mentioned,) the only ones within my knowledge, in which the human spleen was removed.

"One, that of the soldier whose side was laid open by a sabre wound at the battle of Dettingen, (if my memory serve,) the spleen protruding and lying out for some hours in the dirt. It was removed by the surgeon. The man recovered, and seemed to suffer afterwards no inconvenience referrible to the want of the spleen. Mr. Cline used to relate this case.

" A second, that recorded by Dr. O'Brien in his inaugural dissertation.* The case was under his own personal care. The man was a native of Mexico: the spleen lay out for two days before the surgeon was applied to: the bleeding was profuse: the vessels and other connexions were secured by ligature, and the spleen separated completely from the body on the twentieth day of the wound. On the forty-fifth day the man was discharged from the hospital, cured; and observed to some one about this time, that " he felt as well as ever he did in his life." There was bloody urine till the tenth day, (the only bad symptom which occurred during his recovery,) the kidney having most probably received a wound at the time when the side was laid open.

" 5thly. Three cases may be cited, in which the dropsical ovary was rent, probably extensively, from external violence; they are all that have been brought under my notice, and all terminated favourably. For the full authenticity of the following I pledge myself.

" An unmarried lady, with dropsical ovary, was thrown on the ground with violence, from a two-wheeled carriage, and struck the enlarged abdomen with considerable force against a stone which lay by the road side. A large discharge of urine followed; she became permanently freed from her dropsy; and marrying, died with a retroversion of the womb, which could not be replaced. On inspection, the remains of a ruptured ovarian cyst were discovered, retroverting the uterus, which was fixed firmly in the retroverted position, by means of inflammatory adhesions.

" This case, which may be relied on as authentic, gives additional probability to one related by the late Dr. Kissam, of New York, who was a fellow student of Mr. Gaitskell at Edinburgh, and much esteemed for his activity and talent. In this, as in the former instance, the lady had an ovarian dropsy of many years standing, clearly distinguishable through the abdominal coverings. No abscess occurring, for several days afterwards, a trocar and canula were introduced into the peritoneal sack, and twenty-six pints of bloody serum were drawn off; the patient, notwithstanding the double injury from the rent and operation, getting well without any alarming symptoms. (*New England Journal of Medicine and Surgery*, Vol. v. p. 225.)

" The third case deserves notice, especially as corroborated by the two former, which it resembles. There was swelling in the region of the right ovary, equable, smooth and without distinguishable fluctuation; pain shot occasionally in the course of the round ligament down the thigh; the left limb first, and afterwards the right, became œdema-

" * Edinburgh, 1818."

tous; the general health was little impaired. When straining to reach something on a high shelf, the patient felt some part give way within her, and examining herself immediately afterwards, she discovered that the circumscribed tumour was disappeared, and that there was general abdominal swelling in its place. For a length of time afterwards she seemed to be recovering from this injury, and died, with a schirrus of the uterine organs, and not, as appeared, from the accident. *(Idem.)*

"6thly. May be narrated, two cases, in which an opening was made into the abdomen, with a view of extirpating the dropsical ovary.

"In the first, the operation failed completely. The woman had never been tapped, the ovary held about a pailful; a scirrhous piece, as large at least as the hand, not easily removed, was left in the belly; great collapse occurred, directly the ovarian sack was drawn forth, before it was cut into; but the woman lived between eighty and ninety hours afterwards, without the occurrence of peritoneal inflammation, and died, apparently, from the cachexy produced by the dropsy, and for want of reaction in the system and the wound.

"In the second case, the ovarian cyst was extirpated by Dr. Nathan Smith, formerly, I believe, of Connecticut. *(See American Medical Recorder of original Papers and Intelligence in Medicine and Surgery,* No. 17.*)* The sack contained about eight pints; there were no adhesions of extent and importance; the natural connexion of the ovary was as large as a finger, and the patient got well without a bad symptom.* Dr. Nathan Smith is well known to some gentlemen now in London, and would, I have little doubt, if this were deemed necessary for the sake of science, give proof convincing to the most sceptical of the authenticity of this case. These are the only three operations that I at present know of, coming so immediately under my notice, as to justify citation; at the two first I was myself present. I question much whether, in the first operations of lithotomy and amputation, the proportion of recoveries was so great as one in two.

"7thly. May be mentioned five cases of laceration in the womb or vagina, occurring during parturition, all of which were ultimately brought under my personal notice, though in one case only was I present when the accident occurred.

"In the first, the child was born alive by the natural efforts, and the side of the womb was torn longitudinally, where it unites with the broad ligaments; the woman sinking, of consequence, from flooding. I was requested to inspect the body; the rupture of the womb had not even been suspected during her life.

"In the second case, the vagina, or neck of the womb, was lacerated behind, to the extent of a large hand breadth; the peritoneum being laid open, a clot of blood as big as the hand was found, after death, in the abdomen; collapse occurred, the patient never rallying thoroughly, though she lived for thirty-six hours.

"* I quote from my adversaria, as I have not been able to lay my hand on the original in time for the press." 19.

"The third case resembled the former; the woman died collapsed in about thirty-eight hours; there was, however, more reaction than in the former case.

"In the fourth case the womb was torn in front, and the child escaped into the belly; the bladder was not injured. Collapse occurred in this case, and death took place in less than twelve hours.

"The womb was torn in front in the fifth case also; the child as before escaping into the peritoneal sack. I brought this fœtus away by turning; had my hand among the intestines and on the edge of the liver; felt the large arteries in the back of the abdomen, and grasped gently the empty and contracted womb. The child was brought away dead; the woman recovered pretty completely in the course of four or five weeks, but has never been in a state of robust health since. Her name was Casey; she lived near St. George's church, Southwark; and before her recovery was complete, she came, for greater conveniency, into Guy's Hospital. A few months back, (*i. e.* five or six years after the accident;) I made a careful examination, when no traces of cicatrix were discoverable in the vagina, and the mouth of the womb felt perfectly sound and natural, so that there can, I think, be no doubt that the parts had been torn through above." 21.

We have to apologize for the length of the foregoing extract, though it is probably unnecessary; for we think our readers have long been aware that we never quote a passage where the matter can be abbreviated in our own language.

Every one knows the formidable nature of the *Cæsarian section*, and the general fatality of the measure. Our author informs us that, by a friend of the late Dr. Haighton, it has been thrice performed—once successfully, where the abdominal wound was healed completely by the 6th day, the woman being able to stir about in her house on the 13th day. The other two cases were unsuccessful.

From this small collection of facts, *favourable and unfavourable*, our author modestly admits that no *certain* inference can be drawn—though *presumptive* inferences may, to the following effect.

"1st. That smaller wounds of the peritoneum, as in tapping, hernia, &c. do not, in general, induce fatal peritonitis, or other destructive effects; and, therefore, that the common opinion, not perhaps found on paper, but frequently urged in conversation, and apparently operative in practice, I mean, that inflammation in a spot of the peritoneum, will almost invariably diffuse itself over the greater part of it, is probably unfounded in truth.

"2ly. That extensive divisions of the peritoneum are certainly not of necessity fatal, whether by inflammation or otherwise; and *probably* not generally so.

"3ly. That the womb, spleen, and ovaries may be taken away in the mode mentioned, certainly without of necessity destroying life, and *presumptively* without generally destroying it.

"4ly. That the womb, when developed from pregnancy, may be torn open; that the child may escape into the peritoneal sack, among the viscera; and that the mouth of the womb may be torn off, not indeed, so far as these cases may be relied on, without great danger, but twice, in seven instances, without death.

"5ly. And generally, that the peritoneum and abdominal viscera, though very tender in the human body, will, without fatal consequences, bear more injury than, from their modes of practice, the British surgeons, especially, seem disposed to admit.

"6ly. That all the above inferences, from observations on the human abdomen, are in unison with those drawn from observations on the rabbit, the one set of inferences mutually supporting the other; and in this we have a fact corroborative of the principle for which I have contended elsewhere, that observation on the brute and human subject, when made with caution, may, perhaps, be found more in correspondence with each other, than some surgeons are disposed, at present, to admit. A contrary opinion, so far as it is erroneous, must exert a very baleful influence upon the progress of surgery." 24.

As the facts recorded in the paper create a suspicion, in our author's mind, that a "bolder abdominal surgery" would not be unattended with success, he has ventured to solicit the attention of the profession to the following operations—"all to appearance feasible, though by no means all of equal promise"—and none of them to be hazarded except "*in cases otherwise desperate.*"

1st. A division of the fallopian tubes, and even a removal of a small piece of them, so as to render them completely impervious—a fit addition to the Cæsarian section, and, of course, with the view of preventing subsequent impregnation, without destroying the sexual propensities. Dr. B. thinks this operation desirable in those cases of contracted pelvis, where, notwithstanding the excitement of premature labour in the 7th month, it is still necessary to destroy the child, by opening the head. An opening, says he, two fingers broad, might be made above the symphysis pubis, near the linea alba. The fallopian tubes might then be drawn up to this opening one after the other, and a piece of the tube taken away. This operation, he thinks, is much less dangerous than delivery by perforating the head, when the pelvis is highly contracted.

2nd. *Extirpation of the healthy ovaries*—an operation which he admits "can scarcely, in any instance, be necessary," though he thinks, "it would probably be found an effectual remedy in the worst cases of dysmænorrhœa, and in bleeding from inverted uterus. We think this proposition borders on the wild and extravagant.

3rd. *The extirpation of the ovarian cyst in scirrhus, combined with dropsy, or in simple dropsy.* This operation, our author is persuaded, will ultimately come into general use. "And if the British surgeons will not patronize and perform it, the French and American surgeons will." In despite of all that has been written respecting this cruel operation, we entirely disbelieve that it has ever been performed with success—nor do we think it ever will.

4th. *The removal of a large circular piece of the cyst, in ovarian dropsy, when the sac itself cannot be extirpated.*

This proposal is founded on the fact that, as rupture of the ovary has apparently cured the disease, by laying the cyst open, and thus probably inducing inflammation, so advantage might be gained by the operation here proposed, and on a similar principle.

5th. "*The removal of a cancerous womb, when the ulceration first makes its appearance.*"

Such a removal, *above the pubis,* we think extravagant in the extreme.

6th. "*Extirpation of the puerperal uterus.*" Ditto repeated.

7thly. "Should the bladder give way into the peritoneal sack, and I have two preparations of this accident, why should we not lay open the abdomen, tie up the bladder, discharge the urine, and wash out the peritoneum, thoroughly, by the injection of warm water? This operation would secure a chance of life, if the urine had not been extravasated long, say above half an hour." 28.

8th. Small openings with callous edges, through the neck of the bladder into the vagina, are cured in France, it is said, by the actual cautery. "When the opening is large, says Dr. B., it may probably be closed by ligature, without a bad symptom.

10th. Gastrotomy, in case of intus-susceptus.

This operation has been performed, but never with success. See art. Ileus, in the *Dict. des Sciences Medicales.*

We submit our author's proposals to the tribunal of the professional public, without further comment.

II. The second paper in this essay is on the mysterious process of generation. We cannot afford space to detail the experiments made by Dr. Blundell, to ascertain the disputed point, whether or not the accession of the semen to the female rudiments be necessary to the process of fæcundation. The result of Dr. Blundell's experiments is in correspondence with what we should conclude from anatomy, and from common sense—namely, that the seminal accession *is* necessary to the formation of the new animal, though certain changes in the organs of generation (uterus and ovaries) do take place when the said seminal accession is prevented. The following short passage contains the whole result of the experiments.

"On the whole, then, it seems probable, judging from the appearances related, that generation may be carried forward to a certain extent, although the access of the semen to the rudiments be intercepted. Under these circumstances, the young animal cannot be formed, it is true; but corpora lutea may be generated; the wombs may be developed; and the rudiments, if we may judge from the facts already stated, may even be transferred to the uterine cavity by the play of the fallopian tubes." 49.

Before we dismiss this subject, there are one or two physiological points connected with it, which are deserving of notice.

"It has been asserted by some naturalists, that the corpus luteum is an evidence of genuine impregnation. It seems *certain*, however, from the facts related, that this evidence cannot be relied on; for the luteum, in these experiments, was generated under circumstances in which, as the event proved, impregnation was impossible. Indeed, there seems to be little reason for doubting, that the corpus luteum may be produced, even independently of the sexual intercourse, by the mere excitement of desire in a very high degree. Mr. Saumarez has recounted experiments, in his "New System of Physiology," in which the *luteum* appears to have been generated in this very manner. I have now in my possession a preparation (for which I stand indebted to Dr. Cholmeley and Mr. Callaway,) consisting of the ovaries of a young girl, that died of chorea, under seventeen years of age, with the hymen, which nearly closed the entrance of the vagina unbroken. In these ovaries, the corpora lutea are no fewer than four. Two of them, it must be acknowledged, are a little obscure; though an experienced eye, I conceive, would readily detect them. The remaining two are very distinct, and differ from the corpus luteum of genuine impregnation merely from their more diminutive size, and the less extensive vascularity of the contiguous parts of the ovary. In every other respect, in colour and form, and the cavity which they contain, their appearance is perfectly natural, indeed, so much so, that I occasionally circulate them in the class room, as accurate specimens of the luteum upon the small scale." 57.

The above consideration is evidently an important one, in a forensic point of view, and ought to be borne in mind in medico-legal investigations.

The appearances detailed in the experiments, combined with others, afford a plausible proof that the semen sometimes penetrates as far as the ovaries—a point which has been much controverted. In the varieties of human generation, we sometimes meet with extra-uterine pregnancies, where the ovum not only lodges in the tubes, or the peritoneal cavity, but in the ovary itself—a form of disease the most common of its kind.

"Now, if it be true, as I have endeavoured to prove, that the young animal cannot be formed unless the semen have access to the rudiments, it is evident, that in these pregnancies, in which the fœtus is generated among the graafian vesicles, the semen must have made its way up to the ovaries themselves. It must not, however, be too hastily inferred from this, that the semen always penetrates into these remote recesses of the genitals. Facts have been related, which give a shade of probability to the conjecture, that without the contact of the semen, the rudiments may sometimes descend into the uterus; and certainly, although the opinion is not without its difficulties, it is not impossible that they may meet each other there.*" 59.

III. *Transfusion of Blood.* This is the last subject discussed in the volume before us, and occupies a considerable space. We can allot but a limited space for the analysis of it. The subject of transfusion is one on which little can be said in the way of reasoning—all must depend on facts, or actual experiments. We see instances, indeed, where life is lost by the simple issue, from divided or patulous vessels of a certain quantity of the vital fluid. And, as we have no proof that the blood loses its vital properties in the transit from one animal to another, it would appear reasonable, or at least feasible, that a timely recruit of the pabulum of life, in cases of the above nature, might ward off the fatal event. We mean cases of wounded arteries or veins—and uterine hæmorrhage.

"Now, in cases of this kind, (and I might enumerate others which have fallen under my personal notice,) when the patient is gradually sinking, and the bleeding is suspended, there is a fit opportunity for trying the operation of transfusion; and, unless we are prepared, in the face of opposing facts, to deny the utility of the operation altogether, it must, I think, be admitted that it would be used, in such emergencies, with the fairest prospect of preserving the patient's life." 65.

With the view of elucidating this subject, our author made a number of experiments on dogs—draining them of blood till apparent death took place, and then injecting blood from the carotids of other dogs. The time which elapsed between apparent death and the introduction of fresh blood, was various—from half an hour to five minutes. In only one instance (interval five minutes) was resuscitation effected. In this case "the effects were immediate. Shuddering first occurred, then res-

"* Is the transfer of the semen beyond the womb the cause of extra-uterine pregnancy?"

piration, then vascular action, and ultimately complete recovery." From these experiments there can be little drawn that is favourable to transfusion, after asphyxia from hæmorrhage has actually taken place; yet, our author thinks that there is sufficient to bear him out in the assertion that, "in cases of this kind, in which there is no other hope, the operation may deserve consideration." For numerous experiments, ingeniously devised, and skilfully executed, with the view of ascertaining the best modes of transfusion, and how far the blood of animals (differing in species) may be injected into the veins of one another, we must refer to the volume under review, from page 66 to page 136.

We now come to the end of the work, in which is given a very brief account of six cases in which injection into the human veins was attempted.

1. Our author was called to a woman who was dying (as supposed) of loss of blood during placental delivery. The patient had ceased to respire before Dr. B. entered the room. In five or six minutes afterwards, about sixteen ounces of blood were readily procured by venesection (from two men) and thrown into a vein in the arm of the deceased. No signs of resuscitation were observed.

2. A young muscular man, in Guy's Hospital, lost a large quantity of blood, from the bursting of an artery—and appeared for two or three hours afterwards to be evidently sinking from inanition. Our author did not arrive till some minutes after respiration had ceased. Blood was injected; but, with the exception of a single sigh, no signs of returning life were perceptible. There was no difficulty experienced in the transfusion.

3. This was a case of placental hæmorrhage, where the transfusion was commenced before respiration ceased. But the supply was inadequate, and no good effect was the result.

The fourth case was equally unsucessful.

"5. A poor fellow in Guy's Hospital, (his name was Brazier) between thirty and forty years of age, lay at the point of death, in consequence of the extenuation produced by obstinate vomiting, arising, as afterwards appeared, from schirrosity of the pylorus. At the request of Dr. Cholmeley, and the expressed wish of the patient, the late Mr. Henry Cline and myself injected, by means of the syringe, twelve or thirteen ounces of blood into the vein usually laid open in venesection, when no ill symptoms, fairly referrible to the operation, were produced. During the first thirty hours afterwards, there was an increase of the strength, and the man appeared mending; but at the end of this period, he began again to sink into a state of collapse, similar to that which had preceded the operation, and died about fifty-six hours after the injection. Not a single bad symptom occurred when the blood was introduced. Could the operation have been repeated, it is not improbable that his life would have been prolonged."* (p. 78.)

6. This was the case of hydrophobia, about which so much bruit was made in the news-papers, not long ago. Our author remarks respecting Magendie's *imagined* case of hydrophobia as follows :—" respecting the operation of Magendie, I think it better to give no opinion favourable or repugnant, till further information." We were the first in this country to deny the reality of the hydrophobia in Magendie's case—and this opinion is now the general one on the continent.

Our author concludes (and so do we) that " whether it be possible to save a patient, when sinking from hæmorrhage, by injecting blood before respiration ceases, these cases do not enable us to judge."

" But, to conclude. The preceding paper contains all the facts, favourable and unfavourable, which are come to my knowledge, and which seem calculated to help the mind in judging, respecting the operation of transfusion. On perusing them, every one who is in the habit of reflecting, will, of course, form an opinion for himself: having, however, thought a little on the subject, I may be permitted to state my own persuasion to be, that transfusion by the syringe is a very feasible and useful operation ; and that, after undergoing the usual ordeal of neglect, opposition, and ridicule, it will, hereafter, be admitted into general practice. Whether mankind are to receive the first benefit of it, in this or any future age, from British surgery, or that of foreign countries, time, the discoverer of truth and falsehood, must determine." 142.

We part from Dr. Blundell with feelings of the highest respect for his zeal and talents.

" * See in Med. Chir. Trans. vol x. a fuller account of this case."

